# Determining the antioxidant properties of various beverages using staircase voltammetry

**DOI:** 10.1016/j.heliyon.2020.e04210

**Published:** 2020-06-18

**Authors:** W.H. Schilder, E. Tanumihardja, A.M. Leferink, A. van den Berg, W. Olthuis

**Affiliations:** BIOS Lab-on-a-Chip Group, MESA+ Institute, University of Twente, the Netherlands

**Keywords:** Food science, Food analysis, Antioxidants, Wines, Teas, Juices, Staircase voltammetry, Antioxidant index, Half wave potential

## Abstract

Antioxidants are molecules that neutralize reactive oxygen species in the human body, reportedly reducing the risk of cancer and cardiovascular diseases. With multiple dietary products being advertised by their assumed high antioxidant concentration, the need for a proper way of analyzing antioxidant containing beverages becomes apparent.

In this research, the antioxidant nature of teas, wines and (superfood) juices is investigated using staircase voltammetry (SV). A new parameter is proposed and evaluated to characterize the antioxidant nature, including its antioxidant *capacity* and *activity*: the Antioxidant Index (AI).

AI showed green tea to have the best antioxidant nature of teas and red wine to be a better antioxidant than white wine. Superfoods did not show better antioxidant behavior than non-superfoods. AI proved to be a promising way of investigating the antioxidant nature of beverages.

## 1 Introduction

Antioxidants are molecules that protect the human body from reactive oxygen species (ROS), highly reactive species that can damage lipids, proteins and nucleic acids (Lobo et al., 2010). Two classes of antioxidants are distinguished: enzymatic and low molecular-weight antioxidants (LMWA), with the latter being antioxidants neutralizing ROS directly by donating electrons (Chevion et al., 2000). Although not all research agrees, overall, there are strong indications that antioxidants reduce the risk of cancer and cardiovascular diseases (Devasagayam et al., 2004; Hollman and Katan, 1999; Shahidi, 1997).

The disease-reducing properties of antioxidants can be explained by the principle of oxidative stress, which is associated with the hereabove mentioned diseases. Oxidative stress occurs when the ratio between ROS and antioxidants is shifted (Birben et al., 2012; Lobo et al., 2010). Since antioxidant species can prevent oxidative stress, a diet with sufficient antioxidants is recommended (Devasagayam et al., 2004; Voedingscentrum, 2018a). With a diet as suggested by health organizations, this is accomplished easily (Voedingscentrum, 2018a).

Nonetheless, mainly in the 90's and zeros and recently with superfoods too, there is a hype in eating dietary products with a supposedly high antioxidant concentration (Voedingscentrum, 2018a, 2018b). Multiple products are known for their antioxidant properties: fruits, that contain high concentrations of the antioxidant ascorbic acid (AA) (Duthie et al., 2006), tea, containing polyphenols including catechins (Cabrera et al., 2006; Gadow et al., 1997; Łuczaj and Skrzydlewska, 2005) and wine, whose antioxidant nature is mainly ascribed to phenolics and AA (Kilmartin et al., 2001). Moreover, dietary products called superfoods are believed to be highly antioxidant (Voedingscentrum, 2018b). Multiple companies advertise their products by highlighting their assumed antioxidant nature. Thus, versatile methods in determining antioxidant properties of food products can be beneficial to test the advertised claims.

The antioxidant nature of various products has been investigated using multiple techniques (Prior and Cao, 1999). Among them is cyclic voltammetry (CV), an electrochemical method that has shown to be a reliable method for quantification of LMWA (Arteaga et al., 2012; Chevion and Chevion, 2000; Shlomit Chevion et al., 2000; Kilmartin et al., 2001). This study proposes the use of another voltammetric method, staircase voltammetry (SV), as a more suitable technique in indicating antioxidant properties. SV minimizes the contribution of capacitive current, which arises from the double layer effect at the WE/solution interface. Therefore, SV should measure the Faradaic antioxidant nature of a solution more accurately. To record both CV and SV, a potentiostat setup with three electrodes is used: a working electrode (WE), a reference electrode (RE) and a counter electrode (CE).

**Figure 1:**
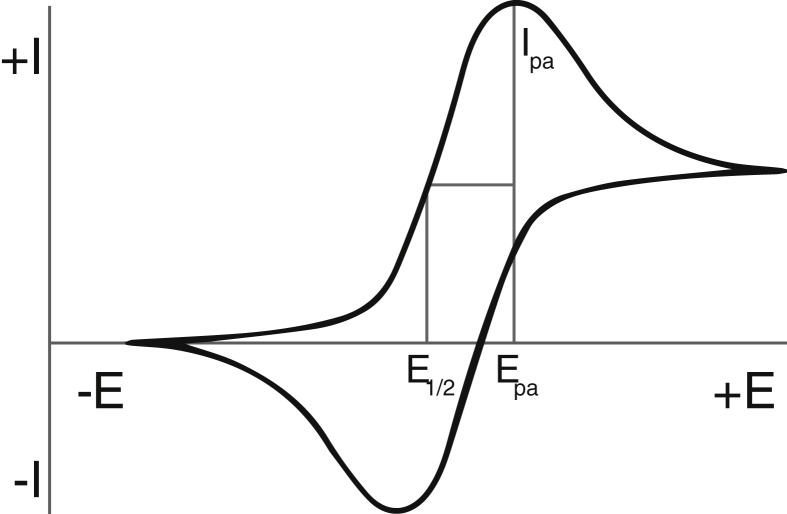
Example CV with E_1/2_, I_pa_ and E_pa_ indicated.

The recorded CV and SV can give information about antioxidant properties in multiple ways (Figure 1). Antioxidant activity can be established by the half-wave potential (E_1/2_) of the oxidation peak, the potential at half the anodic peak current (I_pa_). Antioxidants with low E_1/2_ are stronger electron-donating species (Arteaga et al., 2012; Chevion et al., 2000). However, E_1/2_ alone does not take into account the amount of electrons that can be donated by antioxidant species and thus the concentration of LMWA. I_pa_ is an indication for this concentration and, therefore, antioxidant capacity, but is not as accurate as taking the surface area (Q) under the oxidation peak. Note that the surface area in a CV represents charge Q via the linear relation between applied voltage, V, and time, t: the scan rate. Q is more accurate than I_pa_, since it measures the total amount of exchanged charge expressed in Coulombs, which can be caused by multiple electroactive components in a solution (Chevion et al., 2000). Note that a *lower* E_1/2_ and a *higher* Q are supposed to *increase* the antioxidant nature of the solution.

Still, the best way to determine a solution's antioxidant nature would be a function that includes both antioxidant activity and its capacity, a function of E_1/2_ and Q, respectively. One could present E_1/2_ and Q independently, but they each incompletely reflect the antioxidant nature in a different way. They each represent only one aspect of the antioxidant nature of beverages. Therefore, the following equation is proposed:

(1) $$AI=\frac{1}{2}\frac{Q}{Q_{max}}+\frac{1}{2}\frac{E_{OER}-E_{1/2}}{E_{OER}-E_{HER}}$$

In which AI is the Antioxidant Index, a dimensionless number between 0 and 1. Q_max_ is the maximum Q of oxidation measured in the experiment series in Coulomb, E_OER_ is the standard potential E_0_ of the oxygen evolution reaction (OER) vs. ref (1.031 V vs. Ag/AgCl) and E_HER_ the E_0_ of the hydrogen evolution reaction (HER) vs. ref (-0.199 V vs. Ag/AgCl) (Myrdal, 2006). The lowest antioxidant activity of any beverage is determined by its aqueous background, represented by E_OER_ in the numerator of the second term of Eq. (1). This term is normalized to the difference between the highest and lowest possible oxidation activity, represented by E_HER_ and E_OER_, respectively. Note that a lower E_1/2_ and a higher Q both result in an increased AI, representing an increase in the antioxidant nature of the solution. Both Q and E_1/2_ have been assigned equal weightings.

In this research, the antioxidant nature of green, black and rooibos teas, green ice tea, white and red wine, apple juice, cranberry juice, and two superfoods, cranberry juice and blueberry raspberry juice is investigated using SV. The new proposed parameter AI (Eq.(1)) will be evaluated as a way of determining the antioxidant nature of these beverages. The assumed health benefit is a subject for more research and is no part of the results presented in the current study.

## 2 Materials & methods

**Chemicals** Sulphuric acid (95–98%) was purchased from Sigma-Aldrich and potassium nitrate (>99.5%), which was used as supporting electrolyte, from Fisher Scientific. 1.0, 0.3 and 0.05 μm Alpha Micropolish® II deagglomerated Alumina from Buehler were used for WE polishing. Propanone (100%, technical grade) and ethanol (100%, technical grade) were from Boom. All chemicals were used as received.

**Instruments** SV was recorded using a SP300 potentiostat (Biologic Science Instruments, France). A beaker (the cell) was equipped with a 7.07 mm^2^ glassy carbon WE (CH Instruments, USA), a Ag/AgCl (sat'd KCl) reference electrode (RE) and a 240 mm^2^ Pt counter electrode (CE). Glassy carbon is suitable for measuring antioxidant properties, because of its wide potential window and chemical inertness (Wang, 2000). Moreover, glassy carbon is the most used WE in similar experiments (Arteaga et al., 2012) and ethanol in wine does not oxidize on glassy carbon (Kilmartin et al., 2001). For WE cleaning, a USC 300D ultrasonic cleaner (VWR, USA) was used. The temperature was recorded with a High Precision Pt 1000 thermometer (VWR, Germany).

**WE Cleaning and Storing** Cleaning of the WE during beverage testing was done before each SV. The WE was polished on rayon microcloth (Buehler) using 1.0 μm (minimal 60 s), 0.3 μm (minimal 60 s) and 0.05 μm (minimal 120 s) micropolish respectively. Subsequently, it was rinsed with demineralized water and blow-dried, after which the WE was sonicated for 10 min in propanone. It was then blow-dried and cleaned electrochemically by cycling it 21 times in 1 M H_2_SO_4_ between -1.0 and +1.0 V with SR 100 mV/s, ending in -1.0 V. The WE was stored in ethanol.

**Sample Preparation** The tested beverages are listed in Table 1. GT, BT and RT samples were made by leaving a teabag (GT&RT: ±1.5 g, BT ±2.0 g) perceiving no mechanical interruptions in 200 mL 373 K demineralized water for 2 (GT and BT) or 3 min (RT), following packaging instructions. Teas were left to cool down in open air. When reaching the room temperature, they were filtered using a 0.22 μm sterile filter unit (Millex GP, Cork, Ireland). Remaining beverages were used as received and kept at room temperature. 20 vol% of 500 mM KNO_3_ was added to the samples to obtain a 100 mM KNO_3_ containing solution.

**Table 1:** Overview of the tested beverages.

Category	Beverage	Abbr.	Details
Teas	Green Tea	GT	Pickwick, Amsterdam, The Netherlands
	Black Tea	BT	
	Rooibos Tea	RT	
	Green Ice Tea	IT	
Wines	White Wine	WW	Chardonnay Viognier, Lindeman's, Western Cape, South Africa, 2017
	Red Wine	RW	Cabernet Sauvignon Merlot, Lindeman's, Western Cape, South Africa, 2017
Juices	Cranberry Juice	CJ	Albert Heijn, Zaandam, The Netherlands
	Apple Juice	AJ	
Superfood Juices	Cranberry Juice	SCJ	Ocean Spray, Mönchengladbach, Germany
	Blueberry Raspberry Juice	SBJ	Healthy People, the Hague, The Netherlands

**Testing Procedure** The cell, containing 10 mL sample, was equipped with the WE, CE and RE and subsequently, the temperature of the sample was recorded. SV was recorded successively for 4 cycles between +1.0 and -1.2 V with v_scan_ 50 mV/s (dE = 0.250 mV, dt = 0.005 s), starting and ending in +1.0 V. All measurements were done in two weeks.

**Data Processing** Surface area, anodic peak potential (E_pa_, see Figure 1), half-width, and I_p_ were calculated thrice using EC-Lab® software from BioLogic by manually constructing the best fitting linear baseline under the oxidation peak. E_pa_ was taken at half the surface area. E_1/2_ was at half I_pa_, following Figure 1.

## 3 Results & discussion

**Figure 2:**
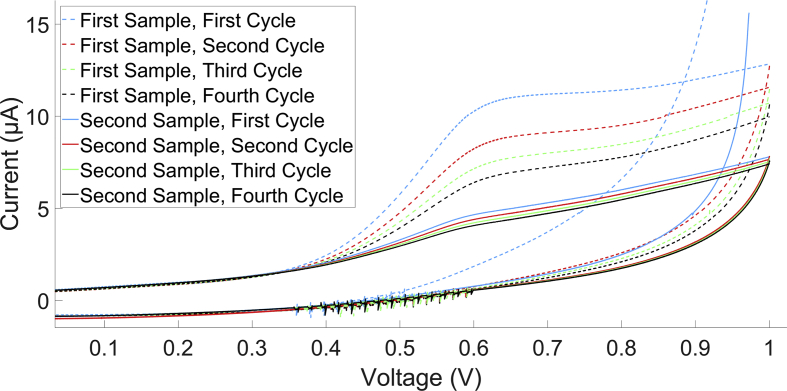
SV of 1.2x diluted SBJ in 100 mM KNO_3_ at room temperature without cleaning in between. Peak current is seen to decrease with each cycle, both at the first run, and at the second run, when a fresh sample was taken. SV were recorded clockwise on a 7.07 mm^2^ glassy carbon WE at v_scan_ 50 mV/s.

**Figure 3:**
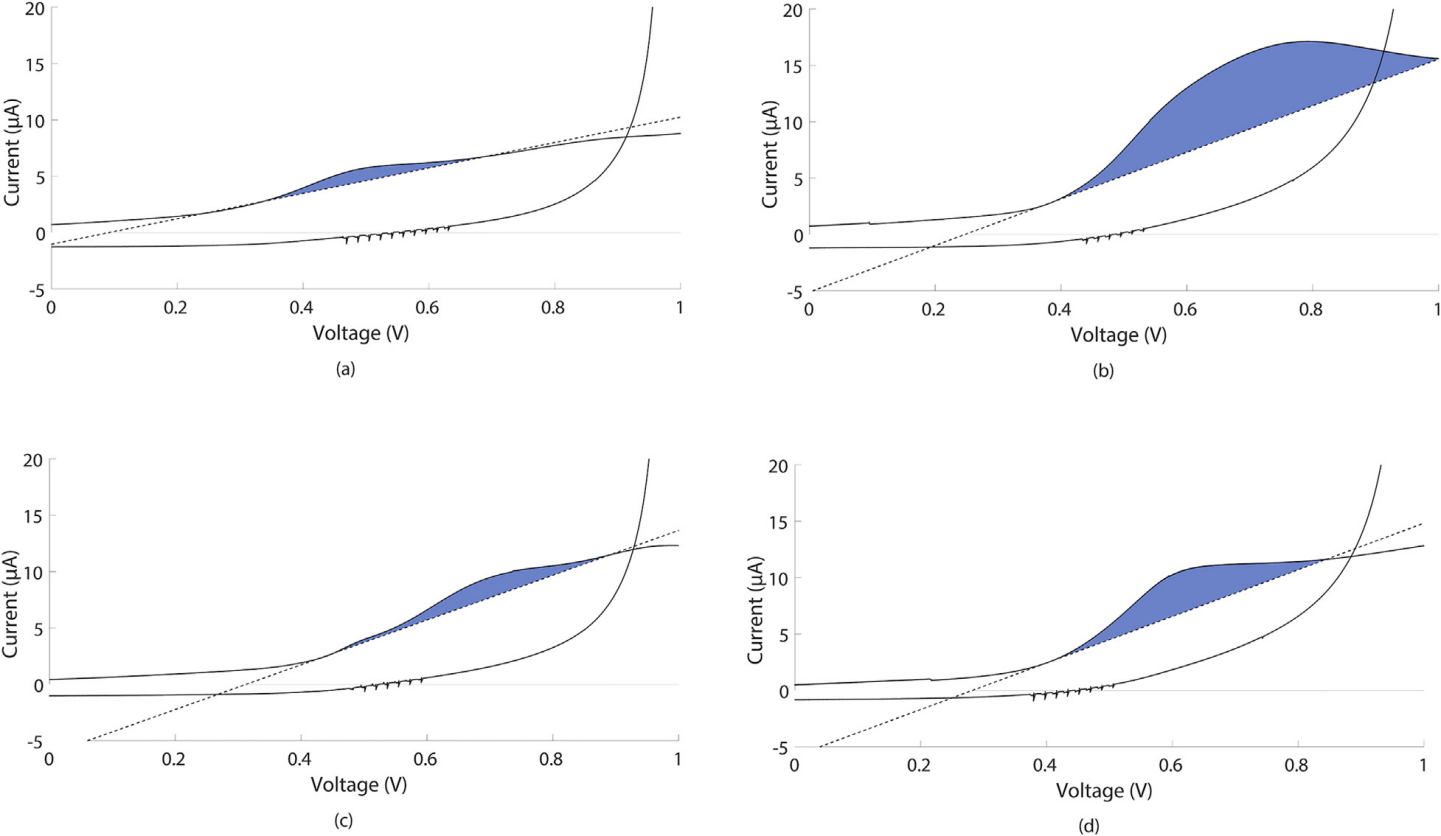
SV of 1.2x diluted a) GT, b) IT, c) RW and d) SBJ in 100 mM KNO_3_ at room temperature and their constructed baseline. The calculated area is marked in blue. SV were recorded clockwise on a 7.07 mm2 glassy carbon WE at v_scan_ 50 mV/s.

In Figure 2, two SV of SBJ are shown. In the first run (First Sample), it was observed that the oxidation peak height decreases in each cycle. This was observed for all samples. Two mechanisms can cause such behavior: adsorption of the sample on the WE, something that was also observed by Kilmartin et al. in their investigation of wine antioxidant properties (Kilmartin et al., 2001), and non-reversible changes in the sample. In case of non-reversibility, refreshing the sample without cleaning the WE should result in a I_pa_ restored to the original value of the first sample. As can be seen in Figure 2, this was not the case. Adsorption to the WE thus explains these observations. Therefore, only the first cycle is analyzed, following Figure 3.

### 3.1 Antioxidant activity and capacity by SV

Typical SV of samples at room temperature (295.0 ± 0.5 K) are shown in Figure 3 (for all SV, see figure SM1). In all SV, spikes can be seen between -0.6 and 0 V and around 0.5 V. This is probably due to a software artifact. The oxidation peaks are indicated in blue. Kilmartin et al. (2001) showed that peaks in a CV of catechin, an antioxidant found in wine, became more prominent when lowering the concentration (Kilmartin et al., 2001). Moreover, these researchers found that only upon dilution, the CV of wines showed increased reversibility. These observations of (non-)reversibility could be valid for the current results, which are also measured at relatively high concentrations. Although a cathodic peak is seen around -0.6 V in the results, this is not an indication of the reversibility of the samples, which should result in a peak at a positive potential (Kilmartin and Hsu, 2003; Kilmartin et al., 2001; Pisoschi et al., 2008). The peak occurring here at -0.6 V is caused by oxygen reduction (Yuan et al., 2014).

The oxidation peaks of GT, BT, WW, and RW occur at a higher potential than can be found in the literature (Kilmartin and Hsu, 2003; Kilmartin et al., 2001). In these studies, more diluted samples were used. Kilmartin et al. (2001) showed that for wine samples, peak separation decreased from 99 mV to 69 mV when diluting from 50 to 400x (Kilmartin et al., 2001). Here, the samples that were used were minimally diluted, which could explain the higher oxidation potentials. Whether a shift in oxidation potential occurs for the other samples too is unclear, since no comparable research was be found.

**Figure 4:**
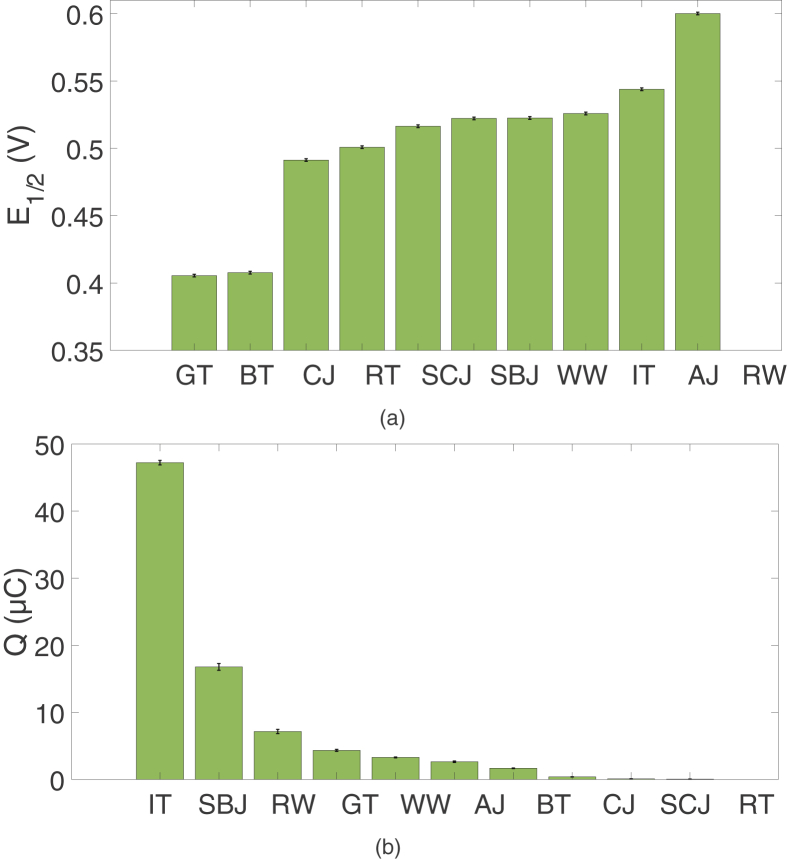
a) E_1/2_ and b) Q of room temperature samples. Low E_1/2_ indicates higher antioxidant activity. High Q indicates higher antioxidant capacity.

Figure 4a shows the E_1/2_ of samples at room temperature. The exact E_1/2_ values can be found in table SM1. GT and BT show the highest antioxidant activity and RW the lowest.

Figure 4b visualizes the Q-values of samples at room temperature. The exact Q-values can be found in table SM1. Noticeable is that Q of IT (47.21 ± 0.33 μC) is almost three times as big as that of SBJ (16.77 ± 0.51 μC). This exceptionally large Q of IT can be possibly explained by the addition of ascorbic acid (AA) as it became clear from the label.

Tea samples showed a ranking based on Q of IT > GT > BT > RT, which for GT and BT conforms to previously performed research using CV (Pisoschi and Petre Negulescu, 2012; Roginsky et al., 2003). IT can be seen as the best antioxidant but is possibly not representative of its product class because of the added AA. RT was with 0.045 ± 0.017 μC, hardly antioxidant. Based on Q-values, RW shows higher antioxidant capacity than WW, concurring with results of Fernandez-Pachon et al. (Fernández-Pachón et al., 2004). Lastly, fruit juices showed a ranking of SBJ > AJ > CJ > SCJ. SBJ, which is advertised to be antioxidative/high in antioxidant, proves to have high antioxidant capacity indeed, while SCJ with just 0.0970 ± 0.0054 μC is hardly antioxidant. Moreover, its Q-value indicates a lower charge exchange than its non-superfood equivalent CJ. Note that E_1/2_ and Q are independent variables: a high E_1/2_ can correspond with a low Q for a certain beverage and the other way around, as becomes clear from comparing Figure 4a with 4b.

### 3.2 Antioxidant Index

**Figure 5:**
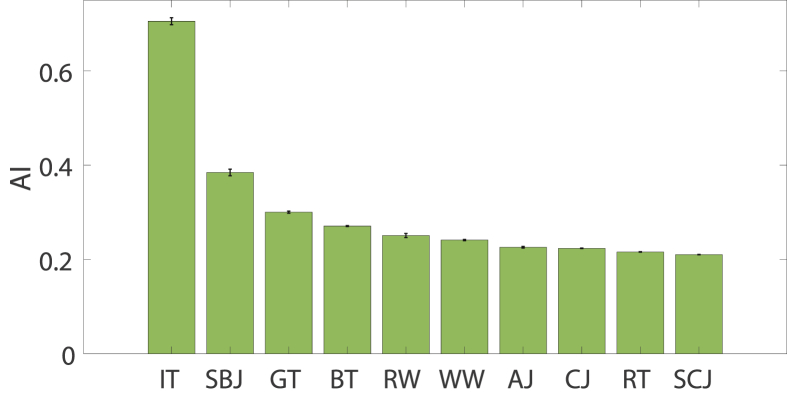
AI of room temperature samples. High AI indicates a better antioxidant.

AI values as calculated by Eq. (1), can be found in table SM1. For this table, Q_max_ of IT (47.21 ± 0.33 μC) is used. In Figure 5, these values are visualized. In comparison to the Q-values in Figure 4B, GT and BT are better antioxidants, while RW shows less antioxidant behavior. This is conceivably accurate as E_1/2_ of RW is 0.2 V higher than that of GT or BT. However, it is debatable how significant E_1/2_ is in comparison to Q. In the current equation, they are given equal weightings. The best way to evaluate this is to compare results to another technique that is suitable for measuring antioxidant nature, such as the 2,2-diphenyl-1- picrylhydrazyl method (DPPH) (Pisoschi and Petre Negulescu, 2012). Weightings can then be adjusted to DPPH results. Moreover, Q_max_ is taken to be that of IT. This way, the value of AI thus depends on what beverages are tested. It would be better to have one substance that results in a high Q_max_ and can be used as a reference in every experiment.

## 4 Conclusion

A novel Antioxidant Index (AI) is proposed as a general measure of antioxidant properties, based on both the antioxidant activity (oxidation potential, E_1/2_) and the antioxidant capacity (amount of charge exchanged).

Based on Q-values, green tea showed the highest antioxidant capacity of teas and rooibos tea the lowest. Red wine had a higher antioxidant capacity than white wine. Superfoods did not show better antioxidant behavior than non-superfoods. Ice tea was shown to be a good antioxidant, possibly due to added AA.

AI showed a similar ranking for teas and wines as Q-values. For superfoods, however, cranberry juice got a much better AI ranking, identical to apple juice, which was not obvious from the Q results. AI has shown to be a promising way of investigating the antioxidant nature of beverages, since it corrects for E_1/2_. However, it needs refinement.

## 5 Recommendations

AI is a first approach to incorporate the antioxidant activity, reflected by the oxidation potential and the antioxidant capacity (current or charge) into one parameter. It should be investigated how the actual scavenging of ROS is improved by either the activity or capacity of antioxidants.

Additionally, it is recommended to search for a compound that can serve as a reference standard for Q_max_. A good option for this would be Trolox, which is also used in the antioxidant determination by Trolox equivalent antioxidant capacity (TEAC) as a reference antioxidant (Zhong and Shahidi, 2015). Moreover, the Antioxidant Index, AI, has to be evaluated by comparing it to an existing analytical technique in order to determine the weightings of the two terms in Eq. (1) referring to antioxidant capacity, Q, and activity, E_1/2_, respectively.

## Declarations

### Author contribution statement

W. H. Schilder: Conceived and designed the experiments; Performed the experiments; Analyzed and interpreted the data; Contributed reagents, materials, analysis tools or data; Wrote the paper.

E. Tanumihardja, W. Olthuis: Conceived and designed the experiments; Analyzed and interpreted the data; Contributed reagents, materials, analysis tools or data; Wrote the paper.

A. M. Leferink, A. Van den Berg: Analyzed and interpreted the data; Wrote the paper.

### Funding statement

This research did not receive any specific grant from funding agencies in the public, commercial, or not-for-profit sectors.

### Competing interest statement

The authors declare no conflict of interest.

### Additional information

No additional information is available for this paper.

## Appendix A Supplementary data

Figure_SM1 Figure_SM1

Table_SM1 Table_SM1

Figure_SM_1_a Figure_SM_1_a

Figure_SM_1_b Figure_SM_1_b

Figure_SM_1_c Figure_SM_1_c

Figure_SM_1_d Figure_SM_1_d

Figure_SM_1_e Figure_SM_1_e

Figure_SM_1_f Figure_SM_1_f

Figure_SM_1_g Figure_SM_1_g

Figure_SM_1_h Figure_SM_1_h

Figure_SM_1_i Figure_SM_1_i

Figure_SM_1_j Figure_SM_1_j
