# Supplementary material for: Determining the antioxidant properties of various beverages using staircase voltammetry
Source: Heliyon. 2020 Jun 18;6(6):e04210. doi: 10.1016/j.heliyon.2020.e04210 (PMC7306597; doi:10.1016/j.heliyon.2020.e04210)
Supplement: Figure_SM1 [file mmc1.docx]

Supplementary Information to “Determining the Antioxidant Properties of Various Beverages Using Staircase Voltammetry”

# Authors

**W.H. Schilder**

Email: w.h.schilder@student.utwente.nl

Universiteit Twente, The Netherlands

**E. Tanumihardja**

Email: e.tanumihardja@utwente.nl

Universiteit Twente, The Netherlands

**A.M. Leferink**

Email: a.m.leferink@utwente.nl

Universiteit Twente, The Netherlands

**A. van den Berg**

Email: a.vandenberg@utwente.nl

Universiteit Twente, The Netherlands

**W. Olthuis**

Email: w.olthuis@utwente.nl

Telephone: +31(0)534892688/5653

Universiteit Twente, The Netherlands


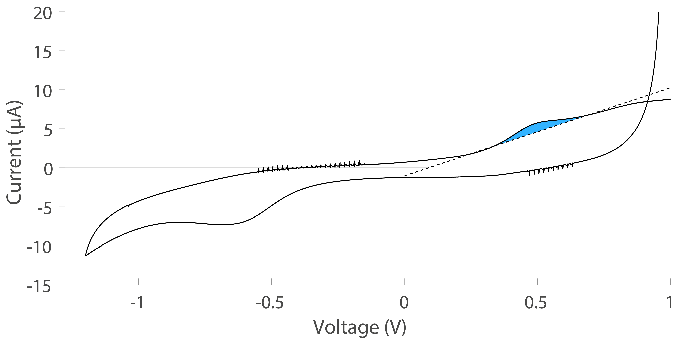

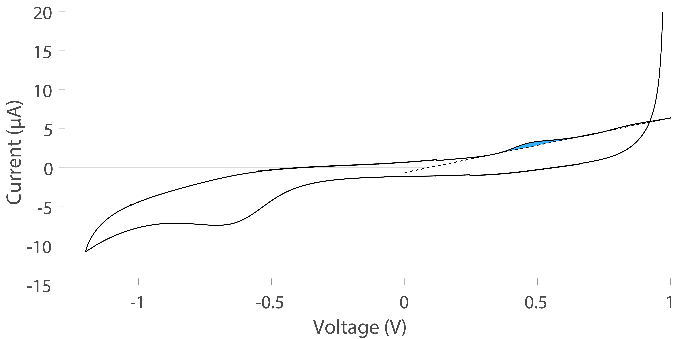


(b)

(a)


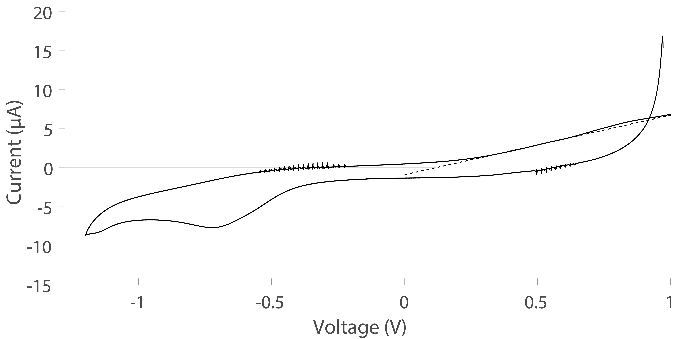

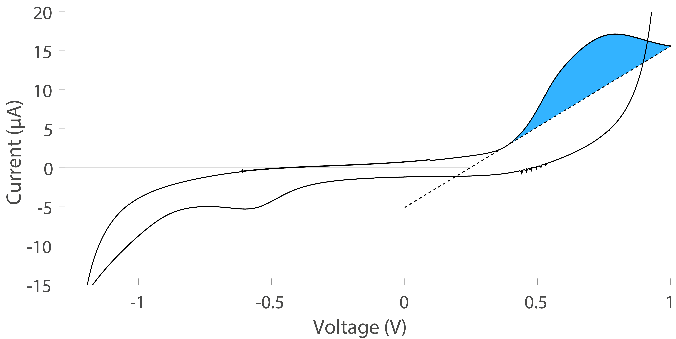


(d)

(c)


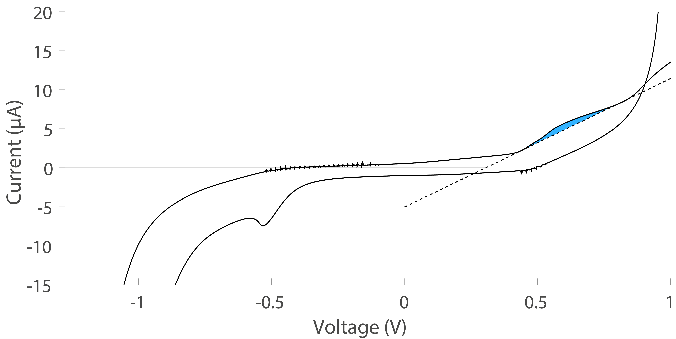

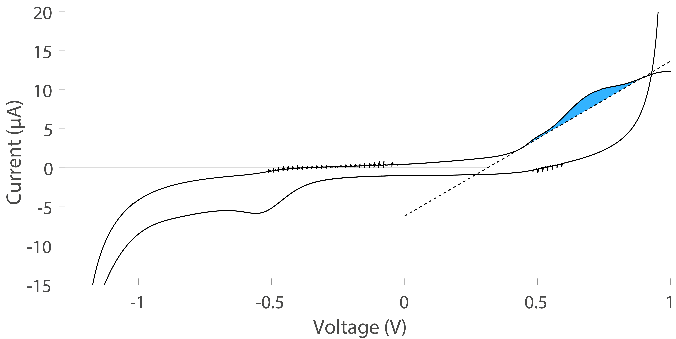

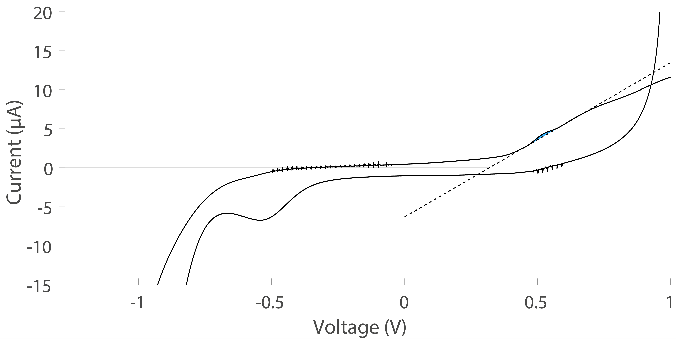

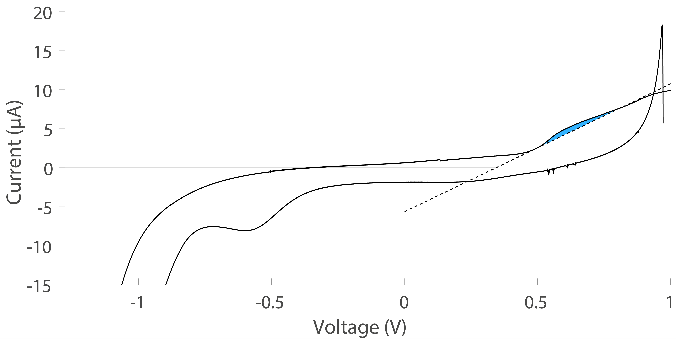

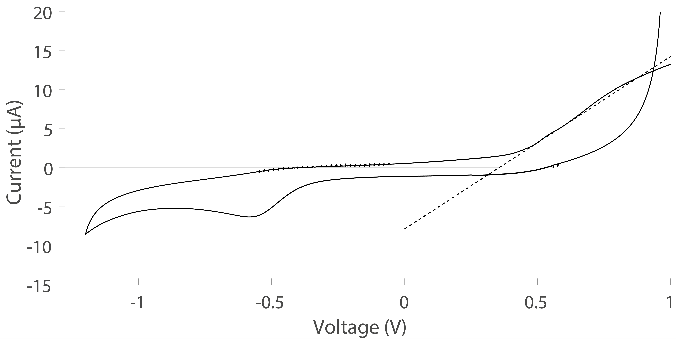

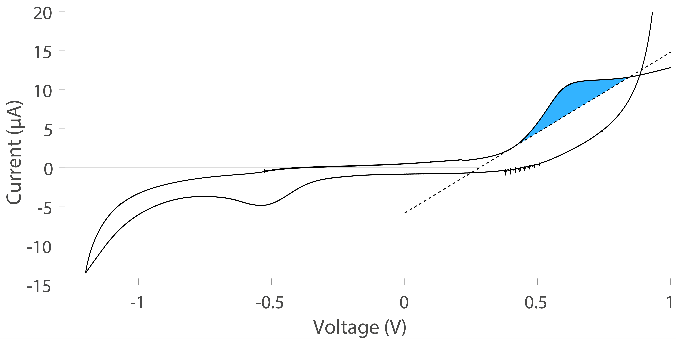


(h)

(g)

(i)

(j)

(e)

(f)

**Figure SM1**: SV of a) GT, b) BT, c) RT, d) IT, e) WW, f) RW, g) CJ, h) AJ, i) SCJ and j) SBJ. All SV were recorded after diluting the sample with 20 vol% of 500 mM KNO_3_ to obtain a 100 mM KNO3 supporting electrolyte background. SVs were all recorded clockwise using a glassy carbon 7.07 mm^2^ WE using a scan rate of v_scan_ = 50 mV/s. The base line for calculating Q (the blue area) is shown in all figures.
