# Supplementary material for: Determining the antioxidant properties of various beverages using staircase voltammetry
Source: Heliyon. 2020 Jun 18;6(6):e04210. doi: 10.1016/j.heliyon.2020.e04210 (PMC7306597; doi:10.1016/j.heliyon.2020.e04210)
Supplement: Table_SM1 [file mmc2.docx]

Supplementary Information to “Determining the Antioxidant Properties of Various Beverages Using Staircase Voltammetry”

# Authors

**W.H. Schilder**

Email: w.h.schilder@student.utwente.nl

Universiteit Twente, The Netherlands

**E. Tanumihardja**

Email: e.tanumihardja@utwente.nl

Universiteit Twente, The Netherlands

**A.M. Leferink**

Email: a.m.leferink@utwente.nl

Universiteit Twente, The Netherlands

**A. van den Berg**

Email: a.vandenberg@utwente.nl

Universiteit Twente, The Netherlands

**W. Olthuis**

Email: w.olthuis@utwente.nl

Telephone: +31(0)534892688/5653

Universiteit Twente, The Netherlands

| **Sample** | **E_1/2_ (V)** | **Q (µC)** | | **AI** | |
| --- | --- | --- | --- | --- | --- |
|  | **Value (std. = 0.001 V)** | **Value** | **Std.** | **Value** | **Std.** |
| **Green Tea (GT)** | 0.405 | 4.33 | 0.15 | 0.3003 | 0.0024 |
| **Black Tea (BT)** | 0.408 | 1.66 | 0.05 | 0.271 | 0.001 |
| **Rooibos Tea (RT)** | 0.501 | 0.045 | 0.017 | 0.2159 | 0.0006 |
| **Ice Tea (IT)** | 0.526 | 47.21 | 0.33 | 0.7053 | 0.0074 |
| **White Wine (WW)** | 0.523 | 3.28 | 0.07 | 0.2412 | 0.0014 |
| **Red Wine (RW)** | 0.600 | 7.14 | 0.32 | 0.2508 | 0.0044 |
| **Cranberry Juice (CJ)** | 0.491 | 0.39 | 0.02 | 0.2236 | 0.0007 |
| **Apple Juice (AJ)** | 0.544 | 2.64 | 0.11 | 0.2259 | 0.0018 |
| **Super Cranberry Juice (SCJ)** | 0.516 | 0.0970 | 0.0054 | 0.2104 | 0.0005 |
| **Super Berry Juice (SBJ)** | 0.522 | 16.77 | 0.51 | 0.385 | 0.007 |

**Table SM1**: E1/2, Q and AI results of all tested beverages.
