# Supplementary figures and images for: Determining the antioxidant properties of various beverages using staircase voltammetry
Source: Heliyon. 2020 Jun 18;6(6):e04210. doi: 10.1016/j.heliyon.2020.e04210 (PMC7306597; doi:10.1016/j.heliyon.2020.e04210)

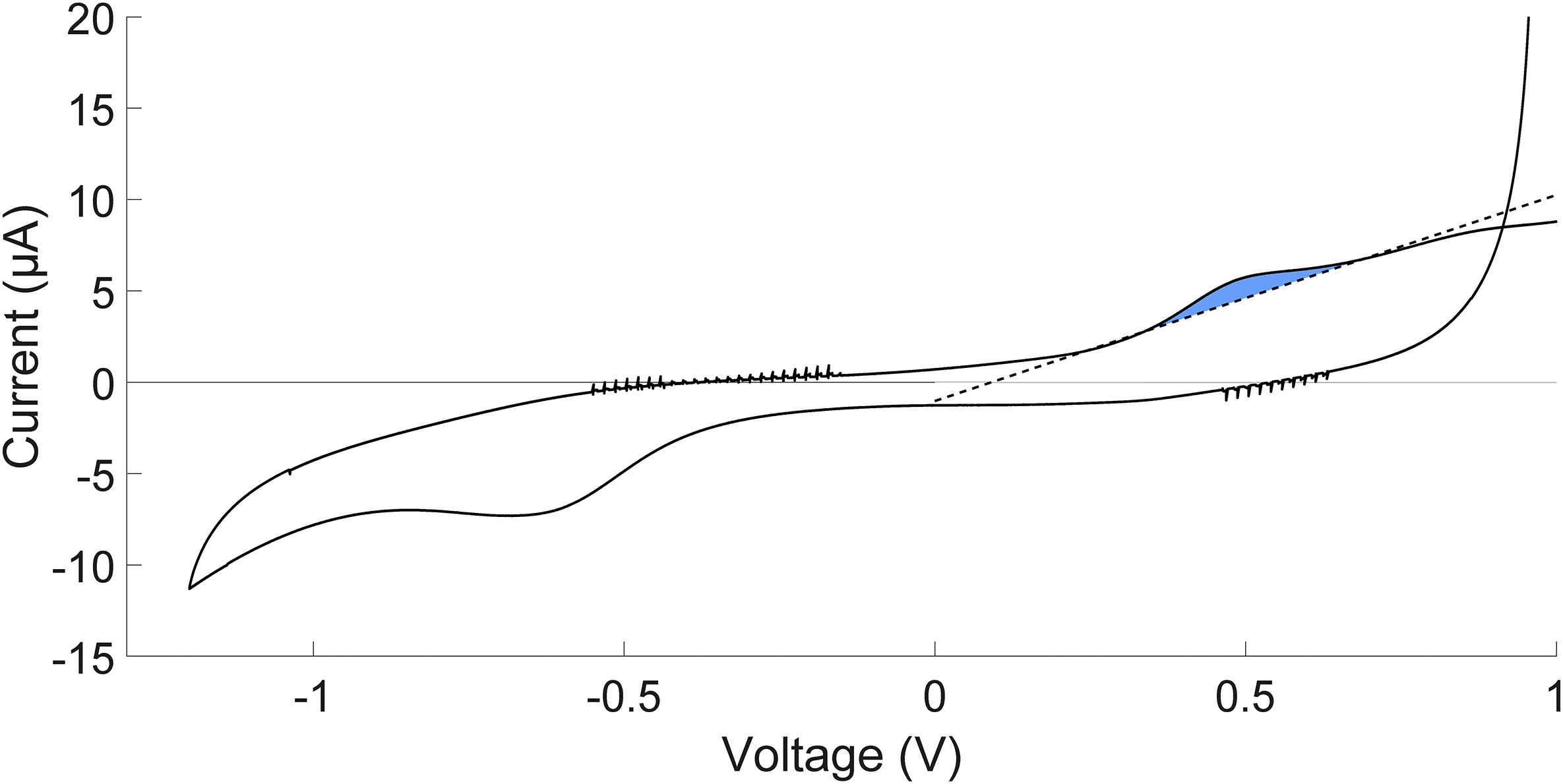

Supplement: Figure_SM_1_a [file figs1.jpg]

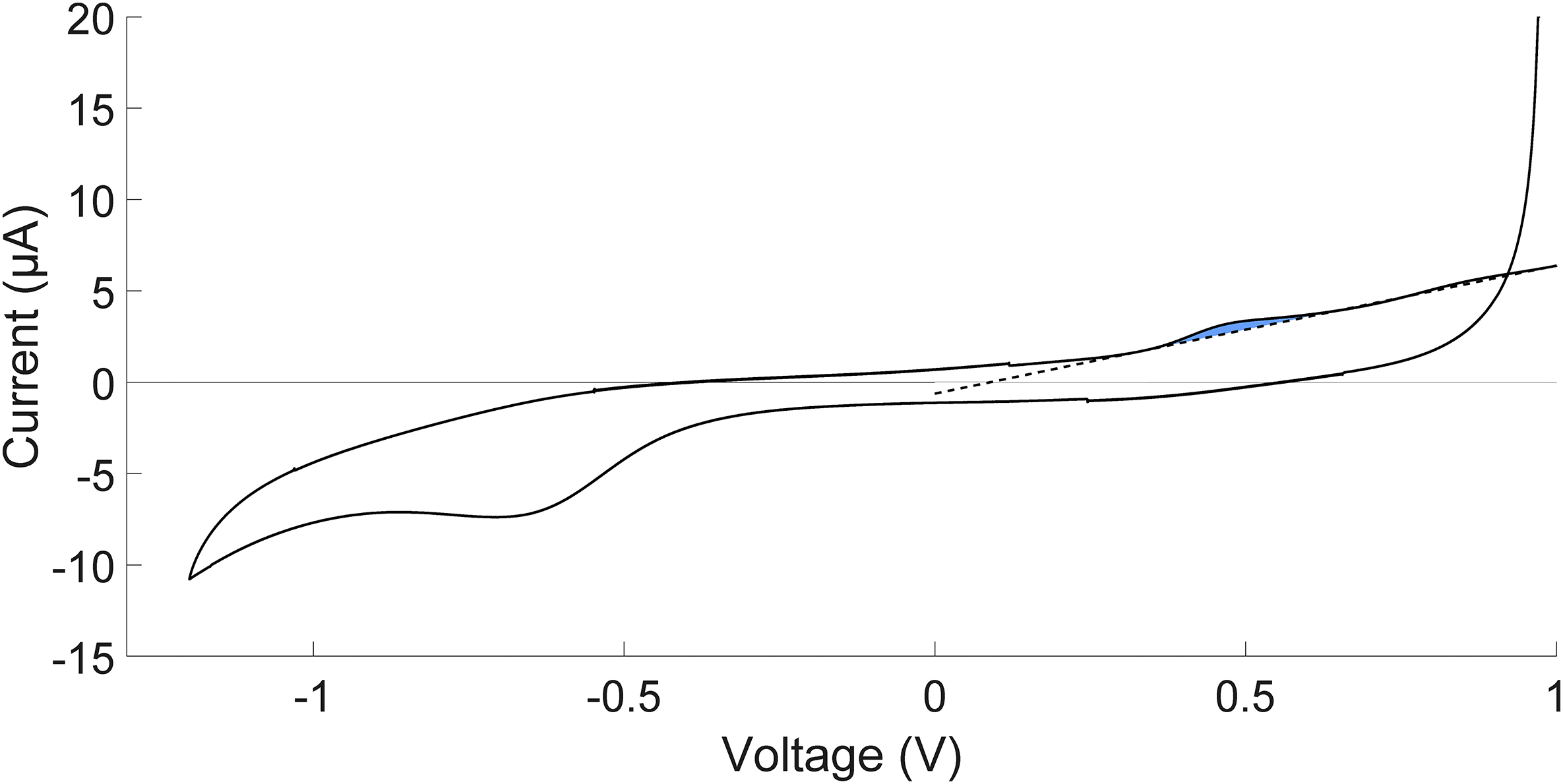

Supplement: Figure_SM_1_b [file figs2.jpg]

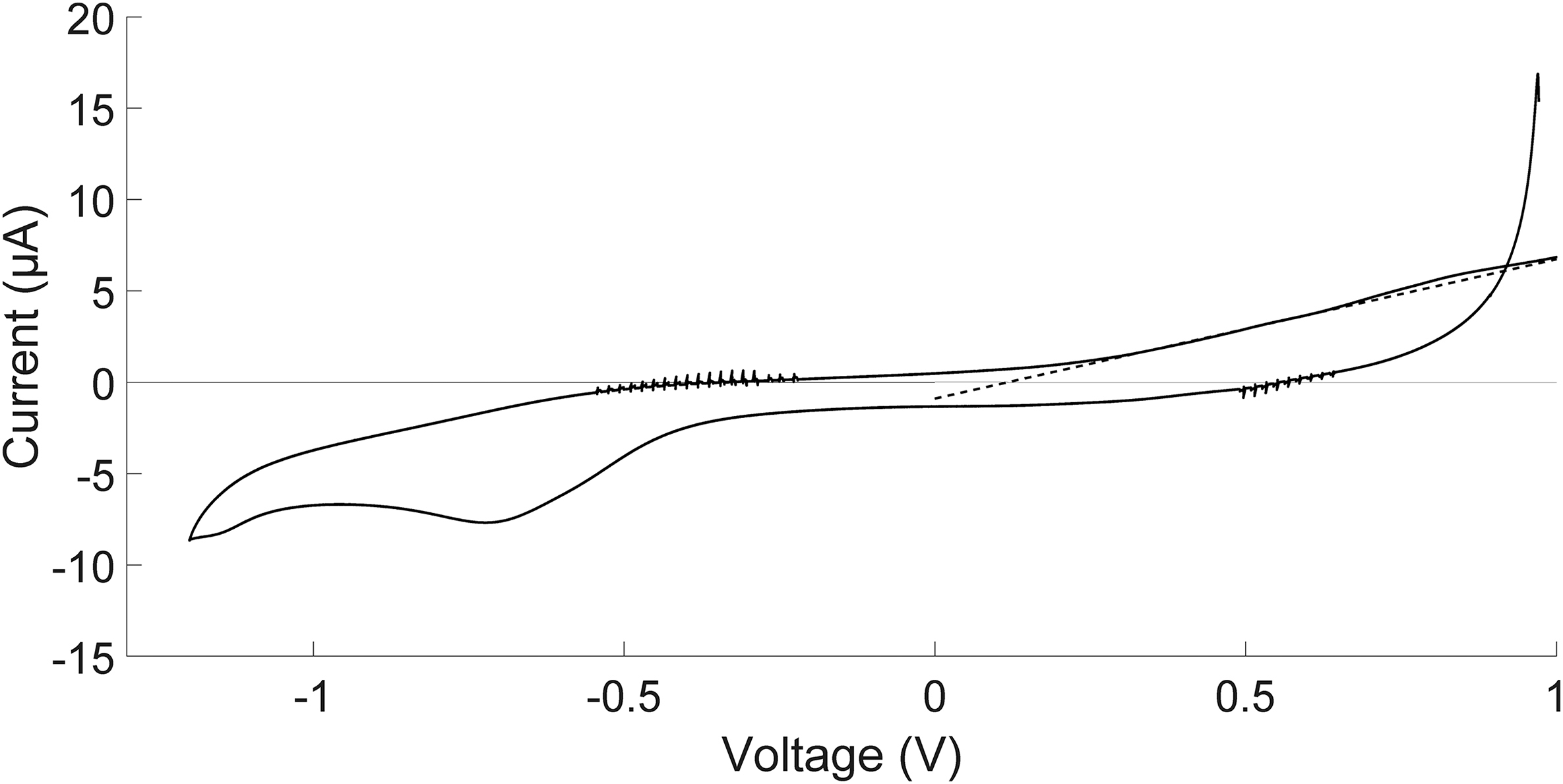

Supplement: Figure_SM_1_c [file figs3.jpg]

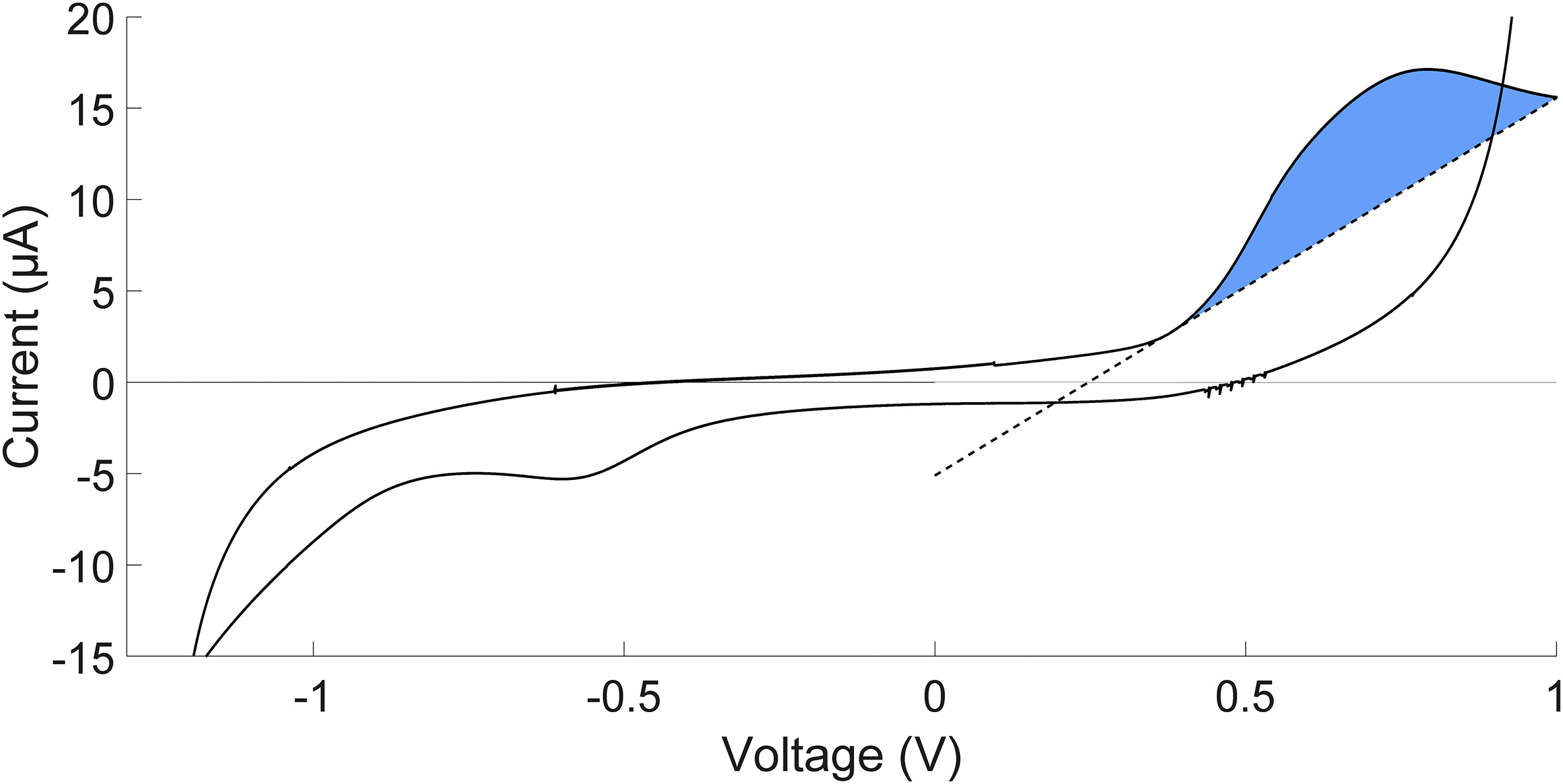

Supplement: Figure_SM_1_d [file figs4.jpg]

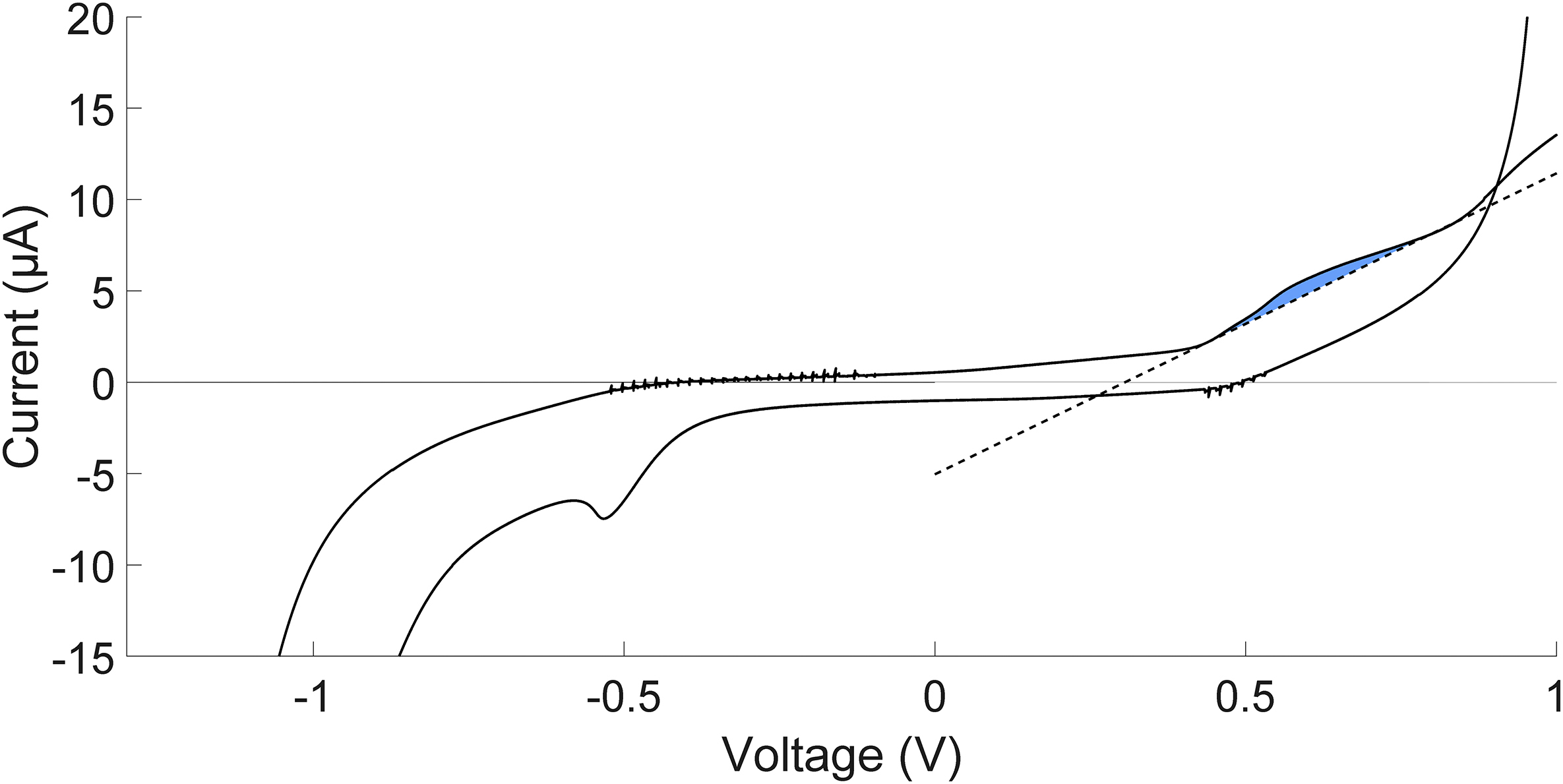

Supplement: Figure_SM_1_e [file figs5.jpg]

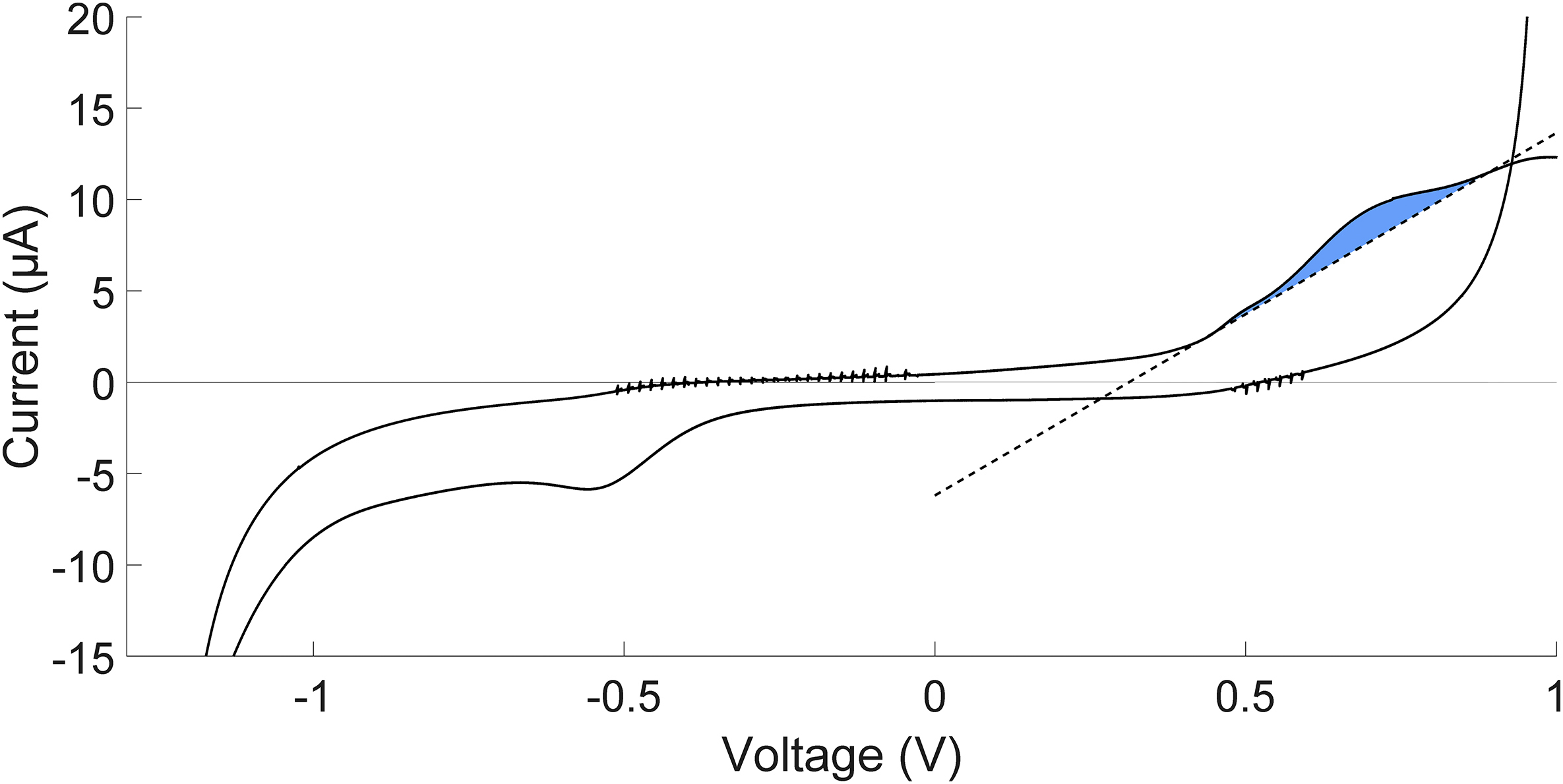

Supplement: Figure_SM_1_f [file figs6.jpg]

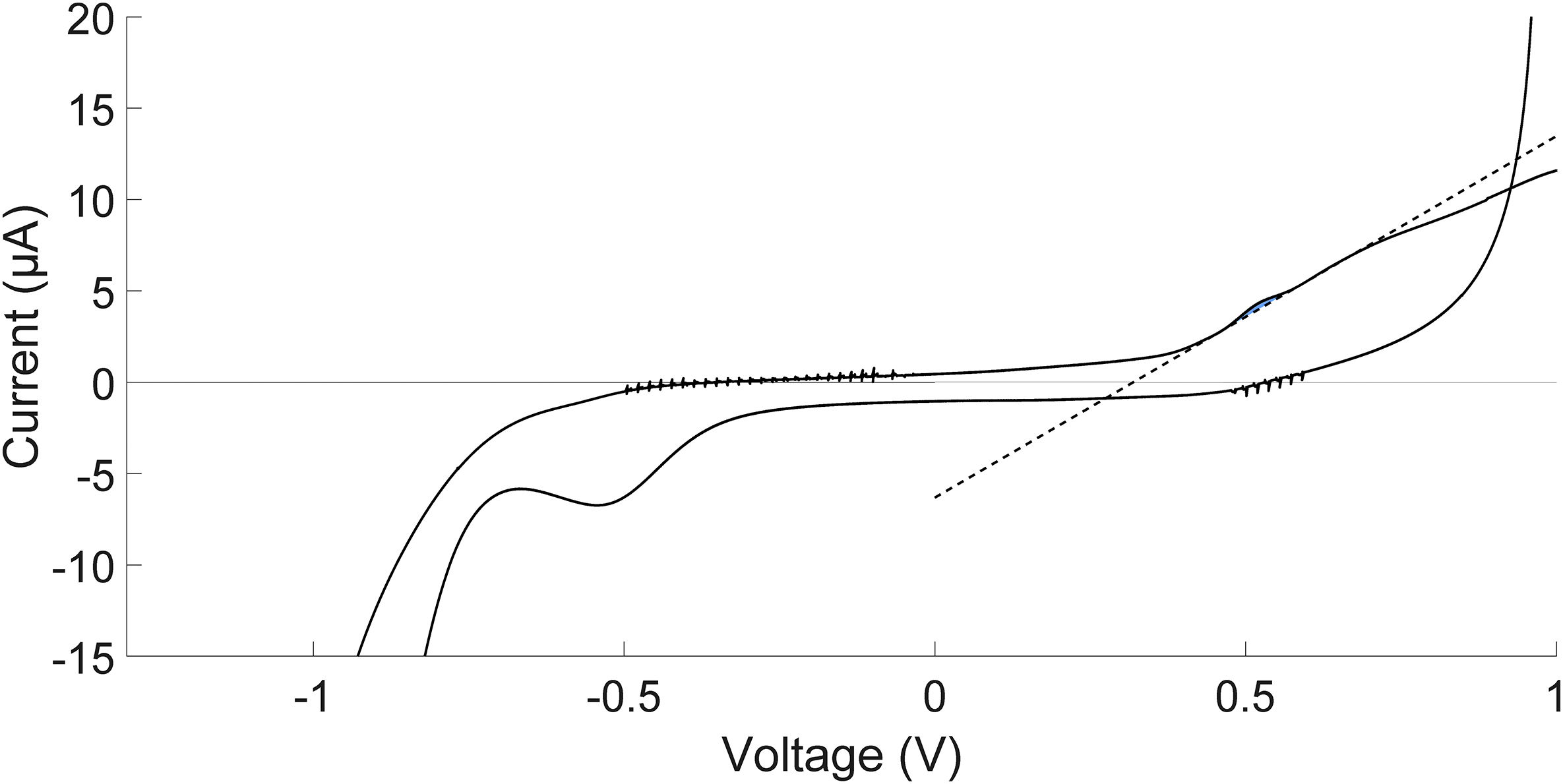

Supplement: Figure_SM_1_g [file figs7.jpg]

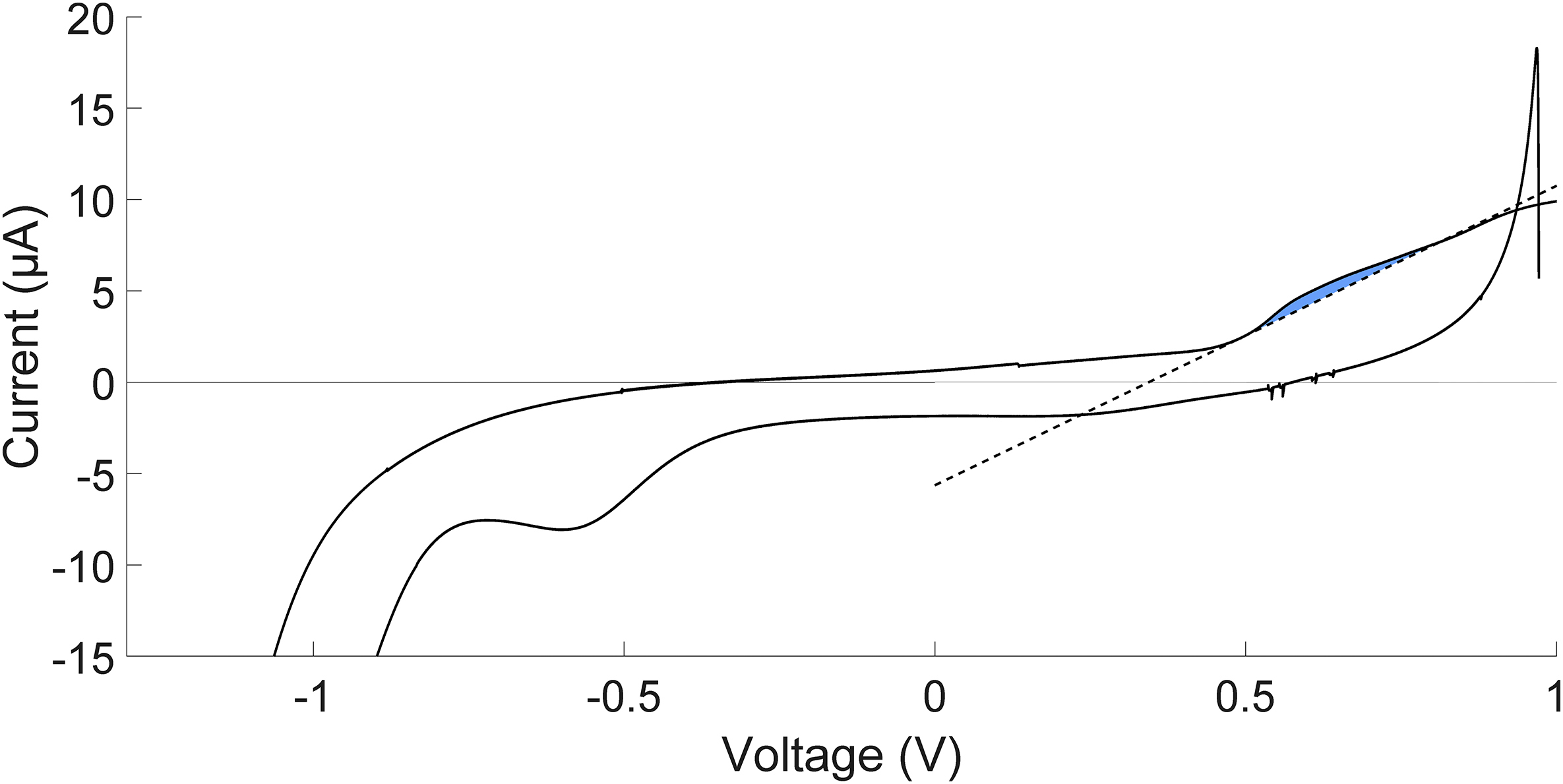

Supplement: Figure_SM_1_h [file figs8.jpg]

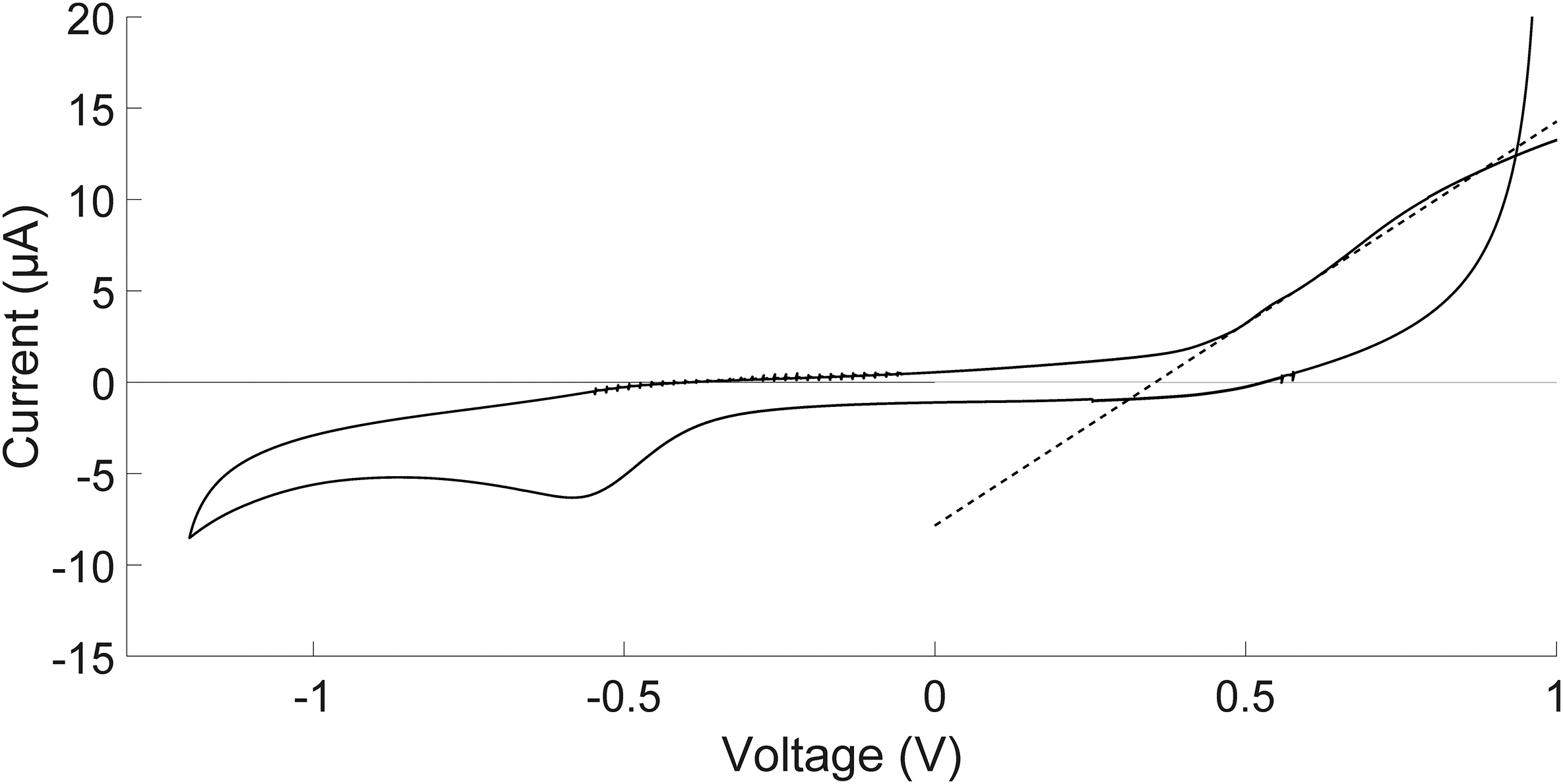

Supplement: Figure_SM_1_i [file figs9.jpg]

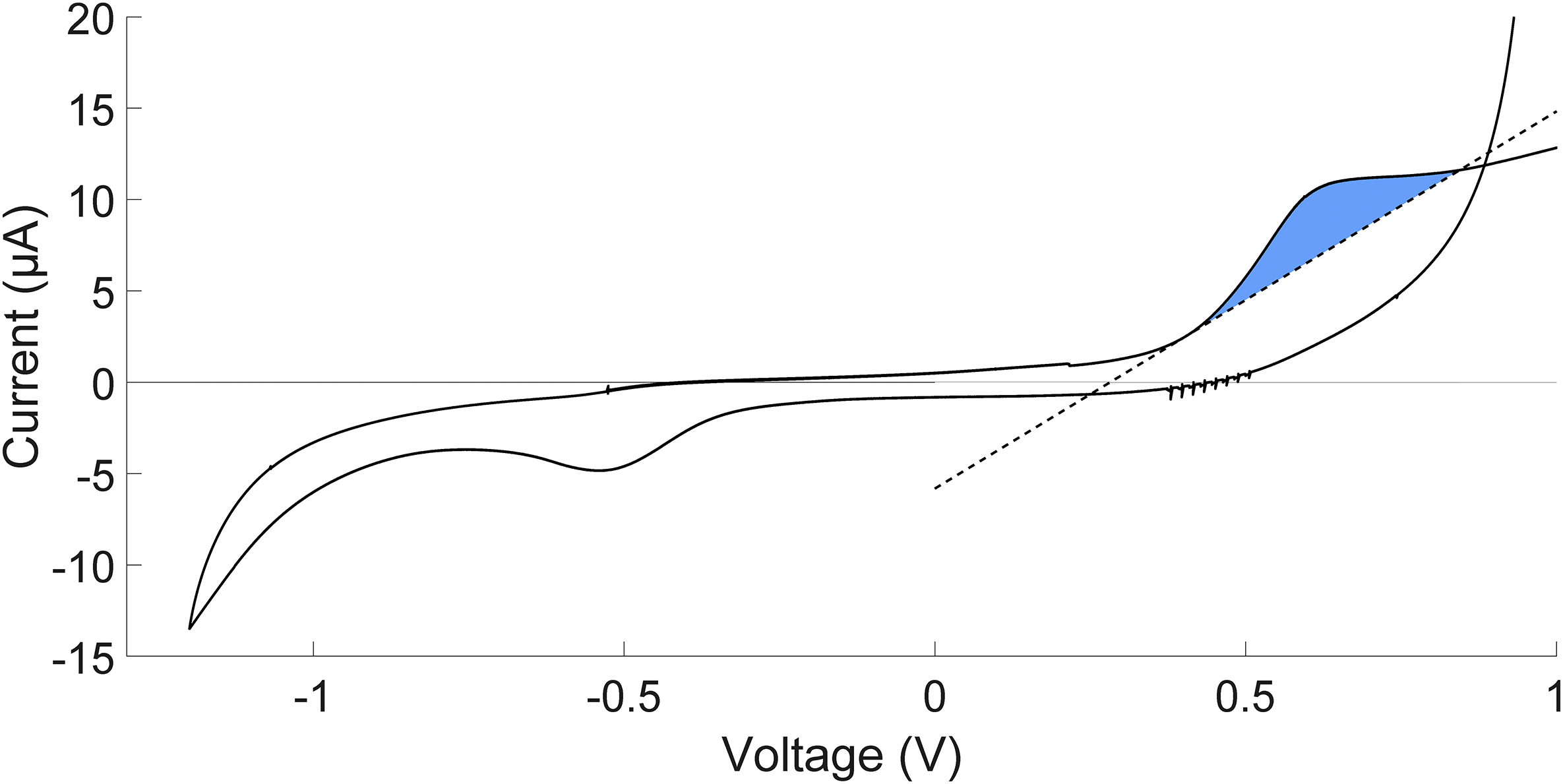

Supplement: Figure_SM_1_j [file figs10.jpg]
